# Comparison of global end-diastolic volume index derived from jugular and femoral indicator injection: a prospective observational study in patients equipped with both a PiCCO-2 and an EV-1000-device

**DOI:** 10.1038/s41598-020-76286-w

**Published:** 2020-11-27

**Authors:** Alexander Herner, Markus Heilmaier, Ulrich Mayr, Roland M. Schmid, Wolfgang Huber

**Affiliations:** grid.15474.33 0000 0004 0477 2438 II. Medizinische Klinik Und Poliklinik, Klinikum Rechts Der Isar Der Technischen Universität München, Ismaninger Straße 22, 81675 Munich, Germany

**Keywords:** Cardiovascular diseases, Mathematics and computing

## Abstract

Transpulmonary thermodilution (TPTD)-derived global end-diastolic volume index (GEDVI) is a static marker of preload which better predicted volume responsiveness compared to filling pressures in several studies. GEDVI can be generated with at least two devices: PiCCO and EV-1000. Several studies showed that uncorrected indicator injection into a *femoral* central venous catheter (CVC) results in a significant overestimation of GEDVI by the PiCCO-device. Therefore, the most recent PiCCO-algorithm corrects for femoral indicator injection. However, there are no systematic data on the impact of *femoral* indicator injection for the EV-1000 device. Furthermore, the correction algorithm of the PiCCO is poorly validated. Therefore, we prospectively analyzed 14 datasets from 10 patients with TPTD-monitoring undergoing central venous catheter (CVC)- and arterial line exchange. PiCCO was replaced by EV-1000, *femoral* CVCs were replaced by *jugular*/*subclavian* CVCs and vice-versa. For PiCCO, *jugular* and *femoral* indicator injection derived GEDVI was comparable when the correct information about *femoral* catheter site was given (*p* = 0.251). By contrast, GEDVI derived from *femoral* indicator injection using the EV-1000 was obviously not corrected and was substantially higher than *jugular* GEDVI measured by the EV-1000 (846 ± 250 vs. 712 ± 227 ml/m^2^; *p* = 0.001). Furthermore, measurements of GEDVI were not comparable between PiCCO and EV-1000 even in case of *jugular* indicator injection (*p* = 0.003). This is most probably due to different indexations of the raw value GEDV. EV-1000 could not be recommended to measure GEDVI in case of a *femoral* CVC. Furthermore, different indexations used by EV-1000 and PiCCO should be considered even in case of a jugular CVC when comparing GEDVI derived from PiCCO and EV-1000.

## Introduction

Several studies demonstrated the usefulness of trans-pulmonary thermodilution (TPTD)-derived global end-diastolic volume (GEDV) and its indexed value (GEDVI), which is adjusted to body surface area (BSA). GEDVI is a static marker of preload which better predicted volume responsiveness compared to filling pressures in a number of studies^1–4^. Furthermore, GEDVI is part of several algorithms with potential to improve outcome^5–7^.

GEDVI can be measured with at least two commercially available devices: the PiCCO and the EV-1000. In general, both devices use similar methodologies and algorithms to obtain the thermodilution curve. Only the derivation of GEDV and extravascular lung water EVLW is slightly different between the two devices: the calculations based on the downslope time used by the PiCCO-system have been replaced by a “proprietary function” of the maximum ascending and descending slopes of the thermodilution curve in the EV-1000^8–10^.

TPTD can be performed using *superior* or *inferior* vena cava access. However, several previous studies (supplementary Table S1) have shown a marked overestimation of GEDVI when performing TPTD indicator injection using a *femoral* venous access due to the additional volume of the vena cava inferior and the prolonged transit time of the cold bolus (supplementary Table S1)^11–15^. Since about 20–35% of all CVC insertions are located in the *femoral* vein^16–18^, the significance of incorrect measurements based on CVC site is substantial. Two recent studies have suggested a correction formula for GEDVI based on data derived from *femoral* TPTD and biometric information^12,13^. Consequently, the manufacturer of the PiCCO device introduced a new software requiring information about the CVC site, and correction for *femoral* indicator injection can be assumed^19^.

So far, only one case investigated *femoral* indicator injection using the EV-1000^13^. Therefore, we compared the agreement of GEDVI sequentially derived by *femoral* as well as *jugular* indicator injection using the EV-1000 and the PiCCO device in 10 patients equipped with both *jugular* and *femoral* venous access.

## Methods

This prospective observational study was conducted in a general ICU between January and March 2017. The institutional review board approved the study (Ethikkommission; Fakultät für Medizin; Technische Universität München; 5384/12). The study was registered at ISRCTN (https://www.isrctn.com/ISRCTN82629192, ISRCTN82629192). The research was performed in accordance with relevant guidelines/regulations. All patients or their legal representatives gave written informed consent.

Patients could be included, if a treating physician not involved in the study decided to perform a change of CVC and arterial line in patients with pre-existing and continuing need for TPTD-monitoring. This decision was made irrespectively of the study according to local standards regarding suspected blood stream infection. Only hemodynamic stable patients without vasopressors or with a constant vasopressor-dosage could be included.

We prospectively recorded 14 datasets from 10 patients with both *jugular* and *femoral* CVC (Multicath 5, Vygon; Aachen, Germany) or with a CVC and a dialysis catheter (Gambro Gam Cath Dolphin; Gambro Hospal GmbH, Gröbenzell, Germany). In general, CVCs or dialysis catheters were inserted in different positions (in the vena cava superior and in the vena cava inferior, respectively). The arterial TPTD curve was generated as previously described^4,20^.

Each dataset consisted of three triplicate TPTDs with the PiCCO-device (GEDVI_PiC_JUG; GEDVI_PiC_FF; GEDVI_PiC_FJ; see Table 1) and two triplicate TPTDs with the EV-1000 (GEDVI_EV_JUG; GEDVI_EV_FEM; see Table 1). All TPTDs were performed with 15 mL of ice-cold saline using the PiCCO-device (Pulsion Medical Systems SE, Feldkirchen, Germany) or the EV-1000-monitor (Edwards Lifesciences, Irvine, USA). A total of 14 datasets were recorded in 10 patients (1 dataset in 7 patients, 2 datasets in 2 patients and 3 datasets in 1 patient).

**Table 1:** Overview about TPTD measurements.

Device	Injection site	Information about injection site	Abbreviation	Comment
PiCCO	*Jugular* CVC	*Jugular* CVC	GEDVI_PiC_JUG	Gold standard for PiCCO
PiCCO	*Femoral* CVC	*Femoral* CVC	GEDVI_PiC_FF	Correct information about CVC. Potential activation of correction for *femoral* indicator injection
PiCCO	*Femoral* CVC	“*Jugular*” CVC	GEDVI_PiC_FJ	Incorrect information about CVC position in order to inactivate a potential correction for *femoral* indicator injection
EV-1000	*Jugular* CVC	Not feasible	GEDVI_EV_JUG	Gold standard for EV-1000
EV-1000	*Femoral* CVC	Not feasible	GEDVI_EV_FEM	Information about CVC-site not requested/feasible
EV-1000	*Femoral* CVC	Not feasible	GEDVI_EV_FEM_CORRECTED	Recalculated with a correction formula as suggested by Saugel, Huber et al.^12^ for *femoral* indicator injection

The measurements with both devices were sequentially performed without an interruption. To avoid a systematic bias due to a potential increase in preload for the last series by repeated TPTDs, the five triplicate TPTDs were performed in random order. The five series of three TPTD-measurements per patient were performed without an interruption, except for changing the position of indicator injection. All patients were stable during the total experimental period of about 15 min. There were no changes in vasoactive substances and fluid supply during the entire period of measurements. The *jugular* venous access was used as the gold standard (Table 1). Indicator injection via *femoral* access *was separately* performed *with* (assumed) activation of a correction for *femoral* measurements and *without* (de-activation of the correction for *femoral* measurements due to selecting the wrong information “jugular/subclavian CVC” in the PiCCO-device equipped with the most recent V3.1 algorithm).

### Objectives and endpoints

This experimental setting allowed the investigation of the following questions:

1. Does femoral indicator injection result in different values for GEDVI, if the correction function in the PiCCO is not activated?
2. Are these differences clinically relevant?
3. Does the latest PiCCO algorithm correct GEDVI for *femoral* injection site?
4. Is this correction appropriate?
5. Does the EV-1000 correct GEDV(I) for *femoral* indicator injection?
6. Are these differences clinically relevant?
7. Do *jugular* measurements of PiCCO and EV-1000 provide comparable results for GEDVI?
8. If the results are non-comparable: is the difference related to different indexations of GEDV?
9. Do *jugular* measurements of PiCCO and EV-1000 provide comparable results for unindexed GEDV in case of *jugular* injection?

### Statistical analyses

Continuous variables were analyzed using Wilcoxon’s matched-pairs test. Bland–Altman analysis was used to evaluate the agreement between variables derived from *jugular* and *femoral* venous catheter sites and to calculate the percentage error. The agreement of classification of GEDVI was analyzed using Kendall’s tau-b coefficient and Fisher’s exact test. The percentage-error was calculated by dividing 1.96*SD by the mean of the compared variables: percentage-error = 1.96*SD/0.5*(GEDVI_A + GEDVI_B) with GEDVI_A and GEDVI_B being two different techniques to measure GEDVI, and SD being the standard deviation of their difference (GEDVI_A – GEDVI_B). A *p* value of *p* < 0.05 was considered significant.

### Sample size calculation

The sample size calculation was based on the findings of the previous study by Saugel et al. with gold-standard GEDVI-values derived from *jugular* indicator-injection of 793 ± 18 ml/m^2^ and significantly higher values of 1094 ± 235 ml/m^2^ for GEDVI derived from *femoral* indicator injection with the PiCCO-device^12^. Based on the online statistical power calculation, sample sizes of n = 5 and n = 10 would provide statistical powers of 90% and 100% respectively (https://www.statisticalsolutions.net/pssZtest_calc.php). Considering a slightly different setting also validating a second device (EV-1000), we chose a sample size of n = 10.

### Ethical approval

The institutional review board approved the study (Ethikkommission; Fakultät für Medizin; Technische Universität München 5384/12). All patients or their legal representatives gave written informed consent.

## Results

### Patient’s characteristics

Table 2 shows the patients characteristics.

**Table 2:** Patients characteristics.

**Based on individual patients (n = 10)**	
Sex (male:female; n (%))	7:3 (70%:30%)
Age (years ± SD)	62 ± 16
*Underlying disease (n (%))*	
Sepsis	4 (40%)
ARDS	4 (40%)
Severe pancreatitis	2 (20%)
APACHE II (score ± SD)	17 ± 7
Height (cm ± SD)	174 ± 7
Weight (kg ± SD)	100 ± 30
**Based on TPTD measurements (n = 14)**	
Measurements under vasopressors	10/14 (59%)
Measurements under mechanical ventilation	14/14 (100%)
Measurements under controlled ventilation (CV)	3/14 (21%)
Measurements under sinus rhythm (SR)	12/14 (86%)
Measurements under SR and CV	3/14 (21%)

### Comparisons of GEDV(I) derived from different TPTD injection sites using the PiCCO or the EV-1000 device

Since most users prefer *indexed* values, we started our analyses with comparisons of GEDVI derived from PiCCO versus EV-1000 using *jugular* or *femoral* TPTD injection.

Based on this approach, we tried to address the following questions:

1. 1Does femoral indicator injection result in different values for GEDVI, if the correction function in the PiCCO is not activated?

**Figure 1:**
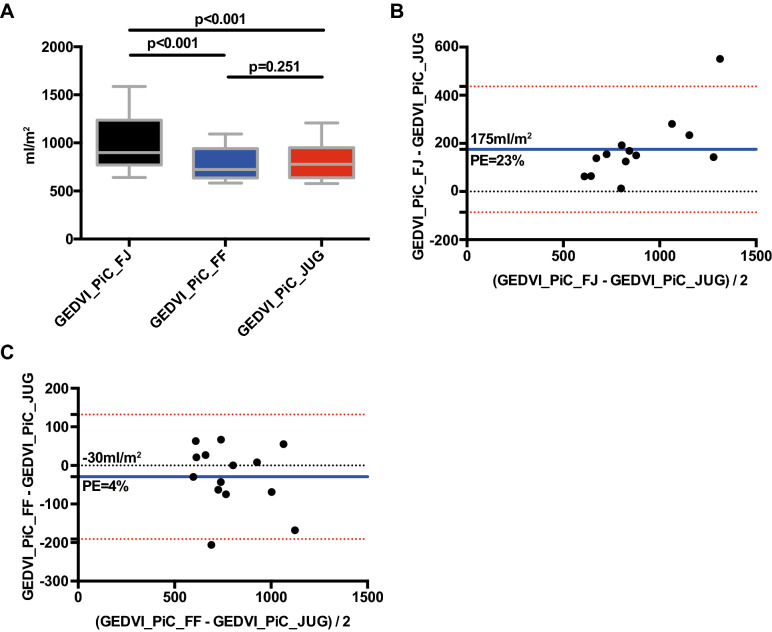
(**A**) Boxplot comparing GEDVI_PiC_FJ, GEDVI_PiC_FF and GEDVI_PiC_JUG. (**B**) Bland–Altman plot comparing GEDVI_PiC_FJ with GEDVI_PiC_JUG. (**C**) Bland–Altman plot comparing GEDVI_PiC_FF with GEDVI_PiC_JUG.

GEDVI_PiC_FJ was markedly higher (980 ± 287 vs. 805 ± 187 ml/m^2^; *p* = 0.001) compared to GEDVI_PiC_JUG with a bias of 175 ± 133 ml/m^2^ and a percentage-error of 23% (Fig. 1A,B).

2. Are these differences clinically relevant?

The uncorrected *femoral* indicator injection resulted in a markedly different distribution of GEDVI classified as decreased (< 680 ml/m^2^), normal (680–800 ml/m^2^) and increased (> 800 ml/m^2^) (Fig. 1B and Table 3). The agreement of classifications of GEDVI_PiC_JUG with those of GEDVI_PiC_FJ was 8 out of 14 (57%; Kendall-tau-b coefficient 0.638; *p* = 0.016). The classifications according to GEDVI_PiC_FJ were significantly different to those according to the gold-standard of GEDVI_PiC_JUG (*p* = 0.016; Fisher’s exact test).

**Table 3:** Comparison of classifications of GEDVI_PiC_fem_FJ and GEDVI_PiC_FF versus GEDVI_PiC_JUG. Consistant results between two groups are marked in bold.

		GEDVI_PiC_fem_FJ (ml/m^2^)			GEDVI_PiC_FF (ml/m^2^)		
		< 680	680 ≤ GEDVI ≤ 800	> 800	< 680	680 ≤ GEDVI ≤ 800	> 800
**GEDVI_PiC_JUG (ml/m**^**2**^**)**	< 680	**2 (14%)**	1 (7%)	1 (7%)	**4 (29%)**	0 (0%)	0 (0%)
	680 ≤ GEDVI ≤ 800	0 (0%)	**0 (0%)**	4 (29%)	1 (7%)	**3 (21%)**	0 (0%)
	> 800	0 (0%)	0 (0%)	**6 (43%)**	0 (0%)	1 (7%)	**5 (35%)**

By contrast, the agreement of classifications of GEDVI_PiC_FF with those of GEDVI_PiC_JUG was 12 out of 14 (86%; Kendall-tau-b coefficient of correlation 0.786; *p* = 0.002), which was not significantly different (*p* = 0.482; Fisher’s exact test; Table 3).

3. Does the latest PiCCO algorithm correct GEDVI for *femoral* injection site?

GEDVI_PiC_FF was comparable to GEDVI_PiC_JUG (776 ± 168 vs. 805 ± 187 ml/m^2^; *p* = 0.251) with a small bias of − 30 ± 82 ml/m^2^ and a percentage-error of 4% (Fig. 1A,C).

4. Is this correction appropriate?

With regard to a low bias, acceptable limits of agreement (Fig. 1C) and classification in the same category for 12 of 14 measurements (agreement of 86%; *p* = 0.482; Fisher’s exact test; Kendal tau-b of 0.685; *p* < 0.001; Table 3) the correction can be considered appropriate.

5. Does the EV-1000 correct GEDV(I) for *femoral* indicator injection?

**Figure 2:**
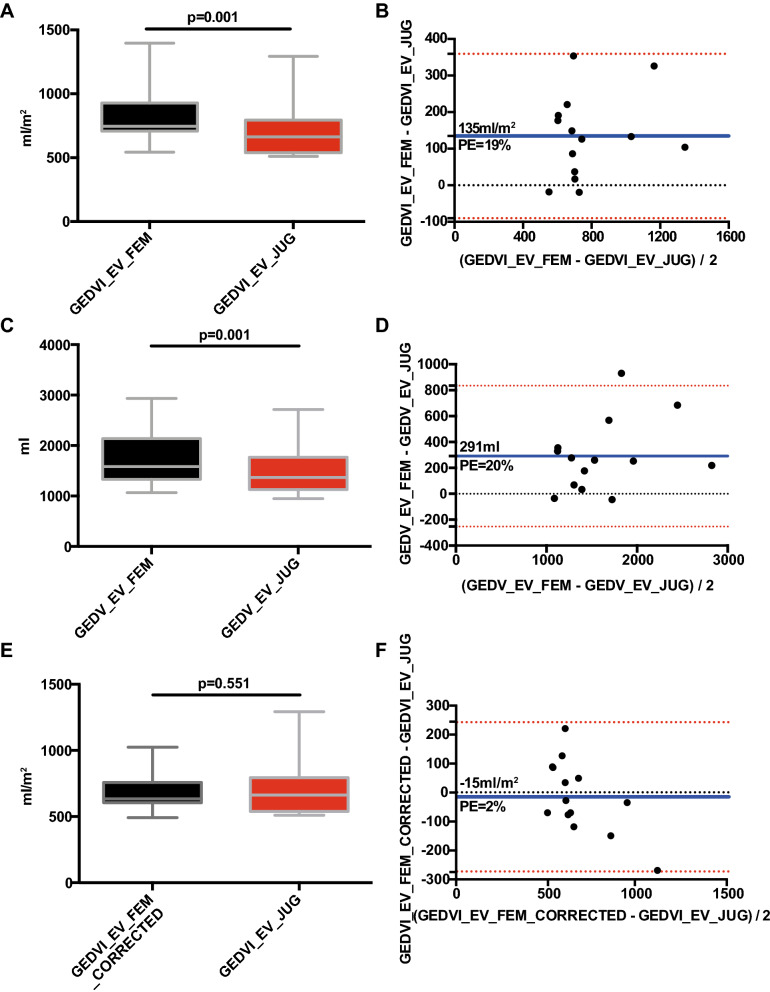
(**A**) Boxplot and (**B**) Bland Altman plot comparing GEDVI_EV_FEM with GEDVI_EV_JUG. (**C**) Boxplot and (**D**) Bland Altman plot comparing GEDV_EV_FEM with GEDV_EV_JUG. (**E**) Boxplot and (**F**) Bland Altman plot comparing GEDVI_EV_FEM_CORRECTED with GEDVI_EV_JUG.

GEDVI_EV_FEM was substantially higher compared to GEDVI_EV_JUG (846 ± 250 vs. 712 ± 227 ml/m^2^; *p* = 0.001) resulting in a marked bias of 135 ± 115 ml/m^2^ and a percentage-error of 19% (Fig. 2A,B). Comparable results were observed for unindexed GEDV. GEDV_EV_FEM was significantly higher compared to GEDV_EV_JUG (1769 ± 574 vs. 1478 ± 483 ml, *p* = 0.001) resulting in a marked bias of 291 ± 278 ml and a percentage error of 20% (Fig. 2C,D).

6. Are these differences clinically relevant?

The lack of correction for *femoral* indicator injection resulted in a markedly different distribution of GEDVI for GEDVI_EV_JUG versus GEDVI_EV_FEM (Table 4).

**Table 4:** Comparison of classifications of GEDVI_EV_FEM and GEDVI_PiC_JUG versus GEDVI_EV_JUG. Consistant results between two groups are marked in bold.

		GEDVI_EV_FEM (ml/m^2^)			GEDVI_PiC_JUG (ml/m^2^)		
		< 680	680 ≤ GEDVI ≤ 800	> 800	< 680	680 ≤ GEDVI ≤ 800	> 800
**GEDVI_EV_JUG (ml/m**^**2**^**)**	< 680	**1 (7%)**	7 (50%)	1 (7%)	**4 (29%)**	1 (7%)	2 (14%)
	680 ≤ GEDVI ≤ 800	0 (0%)	**3 (21%)**	1 (7%)	0 (0%)	**3 (21%)**	1 (7%)
	> 800	0 (0%)	0 (0%)	**3 (21%)**	0 (0%)	0 (0%)	**3 (21%)**

The agreement of the classifications of GEDVI_EV_JUG with those of GEDVI_EV_FEM was only 7 out of 14 (50%). The classification was significantly different compared to the gold standard of GEDVI_EV_JUG (*p* = 0.006; Fisher’s exact test; Kendall-tau-b coefficient of 0.580; *p* = 0.024). Also considering a large bias of 135 ± 115 ml/m^2^ and limits of agreement of 359 and − 90 ml/m^2^ (Fig. 2B), the differences between GEDVI_EV_FEM and GEDVI_EV_JUG are of high clinical relevance. To overcome the difference between jugular and femoral GEDVI measurements we used the correction formula for EV-1000 as suggested by Saugel, Huber et al. for the PiCCO device. The correction of GEDVI_EV_FEM by the formula resulted in GEDVI_EV_FEM_CORRECTED which was not significantly different from GEDVI_EV_JUG (690 ± 148 vs. 712 ± 227 ml/m^2^; *p* = 0.551), with a small bias of − 15 ± 126 ml/m^2^ and a percentage-error of 2% (Fig. 2E,F).

7. Do jugular measurements of PiCCO and EV-1000 provide comparable results for GEDVI?

**Figure 3:**
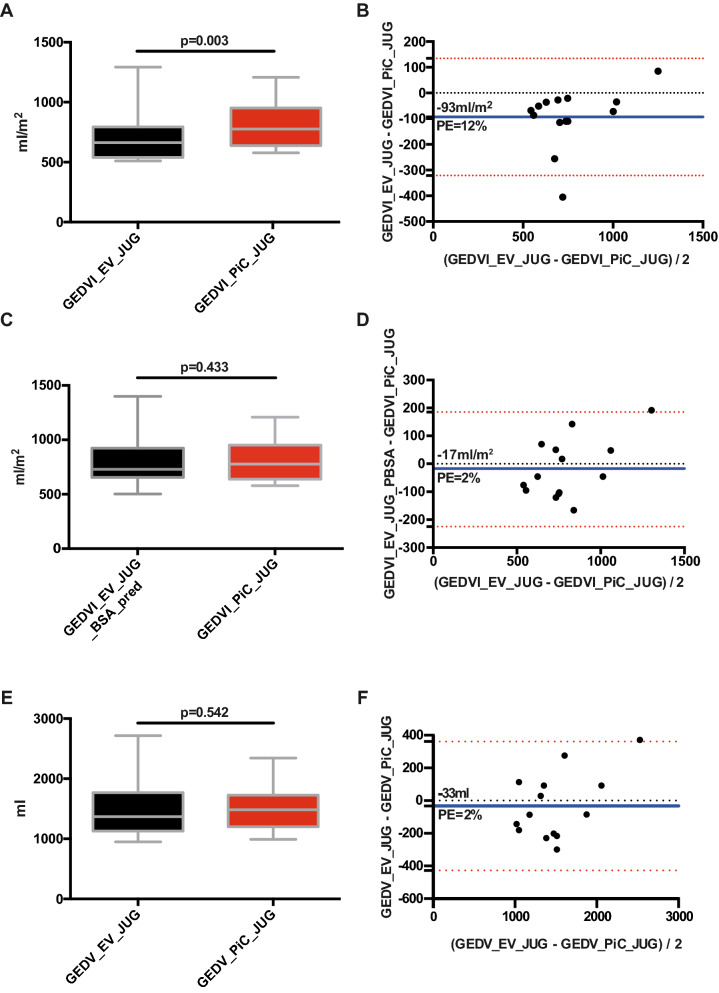
(**A**) Boxplots and (**B**) Bland Altman plot comparing GEDVI_EV_JUG with GEDVI_PiC_JUG. (**C**) Boxplot and (**D**) Bland Altman plot comparing GEDVI_EV_JUG_BSA_pred with GEDVI_PiC_JUG. (**E**) Boxplot and (**F**) Bland Altman plot comparing GEDV_EV_JUG with GEDV_PiC_JUG.

The GEDVI_PiC_JUG was significantly different to GEDVI_EV_JUG (805 ± 187 vs. 712 ± 227 ml/m^2^; *p* = 0.003; Fig. 3A). This resulted in a bias of − 93 ± 116 ml/m^2^, a percentage-error of 12% and limits of agreement of 134 and − 321 ml/m^2^ (Fig. 3B).

Furthermore, in 4 out of 14 cases (29%), GEDVI was differently classified (Table 4). Although the classifications were not significantly different according to Fisher’s exact test (*p* = 0.098), the Kendall-coefficient of correlation was at best moderate (0.560; *p* = 0.026).

Also considering the bias of − 93 ± 116 ml/m^2^, the differences between GEDVI_EV_JUG and GEDVI_PiC_JUG are clinically relevant, in particular in obese patients.

8. If the results are non-comparable: Is the difference related to different indexations of GEDV?

GEDV values for both devices were obtained by multiplication of GEDVI with predicted body surface area (BSA_pred for PiCCO) or with actual body surface area (BSA_act for EV-1000). Table 5 demonstrates that predicted versus actual bodyweight (BW) and predicted BSA versus actual BSA were substantially different for several patients.

**Table 5:** Comparison of BW_act, BW_pred, BSA_act and BSA_pred.

Patient	BW_act (kg)	BW_pred (kg)	BSA_act (m^2^)	BSA_pred (m^2^)
1	120	75	2.37	1.94
2	140	79	2.57	2.02
3	90	61	2.06	1.71
4	90	56	1.97	1.62
5	90	75	2.1	1.94
6	90	71	2.06	1.85
7	160	66	2.63	1.8
8	70	72	1.86	1.89
9	76	74	1.98	1.96
10	75	71	1.9	1.85
Mean ± SD	100.10 ± 30.00	70.00 ± 7.04	2.15 ± 0.28	1.86 ± 0.12

Mean BW_act was significantly different from mean BW_pred (*p* = 0.006, Table 5). Accordingly, the mean values were significantly different for BSA_act versus BSA_pred (*p* = 0.006). This hypothesis was supported by the fact that indexation of GEDV_EV_JUG according to BSA_pred (GEDV_EV_JUG_BSA_pred) was comparable to GEDVI_PiC_JUG (786 ± 240 vs. 805 ± 187 ml/m^2^; *p* = 0.433, Fig. 3C). This resulted in a bias of − 17 ± 106 ml/m^2^, a percentage-error of 2% and limits of agreement of 191 and − 225 ml/m^2^ (Fig. 3D).

Furthermore, the difference (GEDVI_EV_JUG–GEDVI_PiC_JUG) significantly correlated with the difference (BSA_act—BSA_pred) (r = − 0.675; *p* = 0.011).

These findings suggest that the difference between GEDVI_PiC_JUG and GEDVI_EV_JUG is caused by different indexations.

9. Do jugular measurements of PiCCO and EV-1000 provide comparable results for unindexed GEDV in case of jugular injection?

We compared unindexed GEDV_PiC_JUG and GEDV_EV_JUG which were not significantly different (1511 ± 383 vs. 1478 ± 483 ml; *p* = 0.542) with a bias of − 33 ± 201 ml and a percentage-error of 2% (Fig. 3E,F).

With regard to a small bias of − 33 ± 201 ml, a percentage-error of 2%, limits of agreement of 361 and − 427 ml and an acceptable agreement of classifications in 10 out of 14 measurements (71%) (*p* = 0.098; Fisher’s exact test, Kendal tau-b of 0.736; *p* < 0.001; supplementary Table S2), GEDV can be considered comparable between PiCCO and EV-1000 in case of *jugular* indicator injection.

## Discussion

Two previous studies suggested interchangeability of GEDVI derived from the two devices^9,10^. However, both studies did not report on the use of *femoral* CVCs for indicator injection, which results in an overestimation of GEDV(I)^12^. Therefore, we compared the results from patients equipped with both PiCCO and EV-1000 as well as with *femoral* and *jugular* CVCs.

Based on this approach our study shows three main results:

- GEDVI-values derived from *femoral* indicator injection with the EV-1000 are significantly higher compared to GEDVI-values derived from *jugular* CVC indicator injection. GEDVI-values are falsely classified in 50% of measurements when using the EV-1000 with *femoral* indicator injection.
- Surprisingly, GEDVI from *jugular* indicator injection was different between EV-1000 and PiCCO, most probably due to different indexations.
- Finally, the current PiCCO-algorithm appropriately corrects for femoral indicator injection.

At first glance these findings might be surprising, since based on two previous studies in 11 pigs^9^ and 72 critically ill patients^10^, “interchangeability of the two methods” has been claimed^10^.

This is most probably related to the different design of those two studies. In the animal study, all CVCs for indicator injection were placed into the *jugular* vein^9^. No *femoral* indicator injections were performed. Therefore, no conclusions about interchangeability in case of *femora*l indicator injection can be drawn.

In the clinical study by Kiefer and co-workers no details about the CVC-position were given^10^. However, even if *femoral* CVCs would have been used in part in this study, interchangeability of *wrong* GEDV-values can be assumed, since the authors used the old PiCCO-algorithm 8.0.0.6 which was not yet correcting GEDV(I) for *femoral* indicator injection. The newer PiCCO algorithm V3.1 requiring information about the venous catheter site and correcting for *femoral* indicator injection became commercially available in parallel with the publication of that study (2012). Therefore, even in case of *femoral* CVC indicator injection the uncorrected GEDV-values of both devices would have been interchangeable despite a substantial overestimation in case of *femoral* indicator injection, since the old PiCCO-algorithm did not correct GEDV(I) for *femoral* CVC site.

By contrast, our findings of a substantial overestimation of GEDVI by the EV-1000 in case of *femoral* indicator injection are in line with the only one case report addressing this issue^13^. Both studies demonstrate that the slightly different algorithm of the EV-1000 to derive raw GEDV from the thermodilution curve is not capable to correct for *femoral* indicator injection resulting in an increase in the mean transit time due to the additional volume of the inferior vena cava^15,19^. In case of femoral indicator injection the two devices are not interchangeable even in case of use of unindexed GEDV due to the absence of a correction in the EV-1000. Schmidt and co-workers demonstrated that this results in a shift of the thermodilution (TD)-curve to the right, while the curve is otherwise nearly unchanged. This shift to the right is caused by a prolonged time of the horizontal part of the TD-curve, before the first changes in blood temperature induced by the thermo-bolus can be detected by the arterial thermistor. By contrast, the derivation of GEDV in the EV-1000 was not based on the initial horizontal part of the TD-curve, but on the maximum up-slope and on the maximum down-slope of the curve^8–10^. Necessarily, the “proprietary algorithm” of the EV-1000 cannot eliminate the changes of the TPTD-curve due to a prolonged indicator transit time to the thermistor before the “up-slope”.

The more surprising finding in our study was that indexed GEDVI-values were significantly different between PiCCO and EV-1000 even in case of *jugular* indicator injection. Regarding this issue, both previous studies comparing PiCCO versus EV-1000 do not allow conclusions, since both studies were restricted to the analysis of GEDV, but not of GEDVI. A closer look to the manufacturers’ handbooks provides the information that the EV-1000 calculates BSA based on the *actual* bodyweight, whereas the PiCCO algorithm uses *predicted* bodyweight. While the discrepancy of indexation has a limited impact in case of normal weight patients, it results in large differences in GEDVI in obese patients. The finding that 2 out of 14 GEDVI measurements (14%) were classified completely different, i.e. GEDVI_PiC_JUG was elevated, whereas the corresponding GEDVI_EV_JUG was decreased, evidences this. This problem has also been discussed by Beutler and co-workers based on the patient with an actual bodyweight of 220 kg^21^.

While non-correction for *femoral* indicator injection can be considered as a substantial deficiency of the EV-1000, clinically relevant differences between GEDVI derived from PiCCO and EV-1000 should be interpreted cautiously regarding superiority of one approach. A recent large database analysis investigating 3812 TPTD measurements in 234 patients suggests slightly higher coefficients of correlation with unindexed GEDV for BSA_pred compared to BSA_act^22^. Furthermore, the ROC-AUC regarding a decreased GEDV < 1260 ml was larger for BSA_pred than for BSA_act (AUC = 0.842 vs. AUC = 0.733) in this study^22^. Despite these slight differences further investigations are required to optimize indexation as well as normal ranges of GEDV and other haemodynamic parameters.

## Clinical implications and strengths

The findings of our study question the use of EV-1000 to measure GEDV(I) in case of *femoral* indicator injection. Furthermore, in obese patients the differences between the indexations used by PiCCO and by EV-1000 should be taken into account, until more specific and consistent indexations are available^23^. Both findings should be considered to improve the role of GEDV(I) as marker of preload.

## Limitations of the study

This study is limited by a low number of patients and its monocentric design. GEDV(I) alterations over time were not investigated in this study. This is a limitation, since observation of changes over time sometimes facilitates the interpretation of data with inappropriate indexations or questionable normal ranges.

## Conclusions

1. While the last PiCCO-software appropriately corrects GEDV(I) for *femoral* CVC-site, *femoral* indicator injection in the EV-1000 results in a substantial overestimation of GEDVI. Therefore, the EV-1000 could not be recommended to measure GEDVI in case of a *femoral* CVC.
2. Due to different indexations, GEDVI-values are not comparable between PiCCO and EV-1000 even in case of *jugular* indicator injection.

## Supplementary information

Supplementary Information.

## Data Availability

The datasets used and/or analyzed during the current study are available from the corresponding author on reasonable request.

## Abbreviations

BSA: Body surface area
BSA_act: Actual body surface area
BSA_pred: Predicted body surface area
CFI: Cardiac function index
CO: Cardiac output
CV: Controlled ventilation
CVC: Central venous catheter
GEDV(I): Global end-diastolic volume (index)
EVLW(I): Extravascular lung water (index)
GEF: Global ejection fraction
PE: Percentage error
PVPI: Pulmonary vascular permeability index
SR: Sinus rhythm
TPTD: Transpulmonary thermodilution
GEDV(I)_PiC_JUG: GEDV(I) derived from jugular indicator injection measured by PiCCO
GEDV(I)_PiC_FF: GEDV(I) derived from femoral indicator injection containing information about femoral position, measured by PiCCO
GEDV(I)_PiC_FJ: GEDV(I) derived from femoral indicator injection containing information about jugular position
GEDV(I)_EV_JUG: GEDV(I) derived from jugular indicator injection, measured by EV-1000
GEDV(I)_EV_FEM: GEDV(I) derived from femoral indicator injection, measured by EV-1000
GEDVI_EV_FEM_CORRECTED: GEDVI derived from femoral indicator injection and recalculated with a correction formula as suggested by Saugel, Huber et al. for PiCCO, measured by EV-1000
GEDVI_EV_JUG_BSA_pred: GEDV derived from jugular indicator injection and indexed with BSA_pred, measured by EV-1000
