# Supplementary material for: Comparison of global end-diastolic volume index derived from jugular and femoral indicator injection: a prospective observational study in patients equipped with both a PiCCO-2 and an EV-1000-device
Source: Sci Rep. 2020 Nov 27;10:20773. doi: 10.1038/s41598-020-76286-w (PMC7695713; doi:10.1038/s41598-020-76286-w)
Supplement: Supplementary file 1 — Supplementary Information. [file 41598_2020_76286_MOESM1_ESM.docx]

**Comparison of global end-diastolic volume index derived from jugular and femoral indicator injection: a prospective observational study in patients equipped with both a PiCCO-2 and an EV-1000-device**

Alexander Herner, Markus Heilmaier, Ulrich Mayr, Roland M. Schmid, Wolfgang Huber

II. Medizinische Klinik und Poliklinik, Klinikum rechts der Isar der Technischen Universität München, Ismaninger Straße 22, D-81675 München, Germany

**Supplementary Table S1: Studies comparing the PiCCO vs. EV-1000 and/or *femoral* vs. *jugular/subclavian* indicator injection**

|  | **Reference** | **# of subjects and measurements** | **Setting** | **Main result** |
| --- | --- | --- | --- | --- |
| **PiCCO vs. EV-1000** | **Kiefer et al. ^10^** | n=72, 443 TPTDs with EV-1000, no data on CVC position given;  arterial signal re-analyzed by PiCCO | Multicentric study | CO_P_ vs. CO_EV_: r^2^=0,98, mean bias of 0.2 l/min., PE of 9,7 %  GEDV_P_ vs. GEDV_EV_: r^2^=0.93, mean bias 29,4 ml, PE of 11.5 %  EVLWI_P_ vs. EVLWI_EV_: r^2^=0.97, mean bias 36,8 ml, PE of 12.2 % |
|  | **Bendjelid et al. ^9^** | n=11 pigs, 137 *jugular* TPTDs using PiCCO and EV-1000 during inotropic stimulation, hypovolemia, hypervolemia and acute lung injury | Animal study | CO_P_ vs. CO_EV_: r2=0.99, mean bias of 0.2+0.3 l/min.,  PE of 7 %  GEDV_P_ vs. GEDV_EV_: r^2^=0.79, mean bias -11+80 ml, PE of 14 %  EVLWI_P_ vs. EVLWI_EV_: r^2^=0.97, mean bias -5+72 ml, PE of 15 % |
| **GEDVI *jugular* vs. *femoral* CVC** | **Schmidt at al. ^11^** | 11 critically ill patients; n=44 TPTDs | Medical ICU | Overestimation of GEDVI by a mean of 141 ml/m² in case of *femoral* indicator injection. |
|  | **Saugel et al. ^12^** | 48 TPTDs in 24 patients with *jugular* and *femoral* CVC | General ICU | Overestimation of GEDVI by a mean of 241 ml/m² in case of *femoral* indicator injection. Suggestion of a correction formula. Validation of formula in five additional patients. |
|  | **Huber et al. ^9^** | n=1, 10 *jugular* and *femoral* TPTDs using EV-1000 | Case report | *Femoral* GEDVI (p=0.003), PVPI (p<0.001), GEF (p<0.001), and CI (p=0.02) were significantly higher compared to *jugular* measurements. |
| **PVPI, GEF, CFI; *jugular* vs. *femoral* CVC.**  **PiCCO with correction for *femoral* indicator injection** | **Berbara et al. ^20^** | 110 TPTDs in 11 patients | General ICU | Hints from mathematical analyses that PiCCO does not correct PVPI for *femoral* indicator injection. GEDV corrected. |
|  | **Beitz et al. ^14^** | 100 TPTDs in 10 patients | General ICU | Hints from mathematical analyses that PiCCO does not correct CFI, but GEF for *femoral* indicator injection. |
|  | **Huber et al. ^19^** | 54 TPTDs in 25 patients with both *jugular* and *femoral* CVC | General ICU | Direct comparison of *femoral* vs. *jugular* indicator injections demonstrated that new PiCCO-software corrects GEF, but not PVPI for *femoral* indicator injection. |

TPTD: transpulmonary thermodilution; CVC: central venous catheter; CO: cardiac output; PE: percentage error; GEDV(I): global end-diastolic volume (index); EVLW(I): extravascular lung water (index); PVPI: pulmonary vascular permeability index; CFI: cardiac function index; GEF: global ejection fraction

**Supplementary Table S2: Comparison of classifications of GEDV_PiC_JUG and GEDV_EV_JUG**

|  | | **GEDV_EV_JUG**  **(ml)** | | |
| --- | --- | --- | --- | --- |
|  |  | <1260 | 1260≤  GEDV ≤1480 | >1480 |
| **GEDV_PiC_JUG**  **(ml)** | <1260 | **4**  **(29%)** | 0  (0%) | 0  (0%) |
|  | 1260≤  GEDV ≤1480 | 1  (7%) | **3**  **(21%)** | 0  (0%) |
|  | >1480 | 2  (14%) | 1  (7%) | **3**  **(21%)** |
